# Adolescent Access to Federally Funded Clinics Providing Confidential Family Planning Following Changes to Title X Funding Regulations

**DOI:** 10.1001/jamanetworkopen.2022.17488

**Published:** 2022-06-17

**Authors:** Polina Krass, Vicky Tam, Jungwon Min, Isabella Joslin, Lily Khabie, Tracey A. Wilkinson, Sarah M. Wood

**Affiliations:** 1 National Clinician Scholars Program, University of Pennsylvania, Philadelphia; 2 PolicyLab and the Division of Adolescent Medicine, Children’s Hospital of Philadelphia, Philadelphia, Pennsylvania; 3 Department of Biomedical and Health Informatics, Children’s Hospital of Philadelphia, Philadelphia, Pennsylvania; 4 Pediatric Residency Program, Yale University School of Medicine, New Haven, Connecticut; 5 School of Arts and Sciences, University of Pennsylvania, Philadelphia; 6 Department of Pediatrics, Indiana University School of Medicine, Indianapolis; 7 Department of Pediatrics, Perelman School of Medicine, University of Pennsylvania, Philadelphia

## Abstract

**Importance:**

In May 2019, new federal regulations regarding Title X funding were introduced. There has been no formal evaluation of the impact of this regulatory shift as it pertains to minors’ access to services.

**Objective:**

To explore the geography of federally funded clinics providing confidential reproductive care to adolescents following changes to Title X funding regulations.

**Design, Setting, and Participants:**

This retrospective cross-sectional study used a population-based sample of US Census tracts. All clinics participating in the Title X program in August 2018 and August 2020 were included in the analysis. Data were analyzed from January to December 2021.

**Exposures:**

Period, defined as before and after the 2019 Title X rule change (August 2018 and August 2020, respectively).

**Main Outcomes and Measures:**

US Census tracts were evaluated for the availability of confidential family planning care within a 30-minute drive, according to the presence of a Title X clinic or a permissive state law. Census tracts in which minors lost access to confidential care after the rule change were characterized in terms of demographic characteristics. Univariate logistic regression evaluated associations between Census tract characteristics and the odds of losing vs maintaining access to legally protected confidential minor services.

**Results:**

The study included 72 620 Census tracts, accounting for approximately 324 697 728 US residents (99.96% of the population). After the Title X rule change, 1743 clinics in the Title X program left (39.0%) and minors living in 6299 Census tracts (8.7%) lost access to confidential family planning care, corresponding to an estimated 933 649 youth aged 15 to 17 years. Minors living in rural Census tracts (odds ratio [OR], 1.27; 95% CI, 1.18-1.36) and those in the Midwest (OR, 2.41; 95% CI, 2.24-2.60) had higher odds of losing access to care. Minors living in Census tracts with a higher Social Vulnerability Index (OR, 0.51; 95% CI, 0.47-0.55), a larger proportion of Black individuals (OR, 0.34; 95% CI, 0.31-0.37), and/or a larger proportion of Hispanic individuals (OR, 0.45; 95% CI, 0.42-0.49) were less likely to lose access to care.

**Conclusions and Relevance:**

These findings suggest that there were losses in access to legally protected confidential reproductive health services for youth after the 2019 Title X rule change. Although evidence-based Title X guidelines have since been reinstated, state laws that ensure adolescent confidentiality in obtaining family planning services may protect youth from future alterations to the Title X program.

## Key Points

**Question:**

How did minors’ access to clinics providing confidential family planning services change after Title X funding restrictions were introduced in 2019?

**Findings:**

In this cross-sectional study of 72 620 US Census tracts accounting for more than 324 million individuals, following the 2019 Title X rule change, 8.7% of Census tracts lost a clinic that had previously provided confidential reproductive care for minors. An estimated 933 649 youth aged 15 to 17 years lived within a 30-minute drive of these clinics.

**Meaning:**

These findings suggest there were losses in access to confidential reproductive health services for youth after the Title X rule change.

## Teaser

This cross-sectional study evaluates changes in adolescent access to confidential family planning services at federally funded clinics following changes to Title X funding regulations.

## Introduction

Adolescents are more likely to obtain appropriate treatment and preventative care when confidentiality for sexual and reproductive health services is assured.^1,2,3,4,5,6^ Federal family planning funding, with an annual budget of more than $286 million dollars distributed through the Title X program, serves a key role in guaranteeing access to confidential reproductive health care for minors.^7^ At Title X–funded clinics, all minors are legally permitted to consent for and access free or low-cost confidential services. Prior legal cases have upheld that requirements for confidentiality within this federally funded program override state law.^8^ Thus, even in states where laws do not explicitly guarantee minor protections for consent and confidentiality, minors are still able to consent for and access confidential services at Title X–funded clinics, and, as a result, can benefit from the improved sexual and reproductive health outcomes associated with confidential care.^3,5,6^

In May 2019, new federal regulations regarding Title X funding were introduced, colloquially referred to as “the domestic gag rule.” In March 2020, these regulations were fully implemented.^9^ Key policy changes included (1) prohibiting referrals for abortions; (2) requiring abortion services to be physically and financially separated from Title X services (Title X funding has never been allowed to pay for abortions); (3) lifting requirements for nondirective pregnancy options counseling, meaning that clinicians were no longer required to provide unbiased information on all pregnancy options; and (4) permitting clinics to limit types of contraceptive services delivered for reasons of conscience.^9,10,11,12^

In response, many previously Title X–funded clinics publicly withdrew from the program to preserve their existing practices, including almost all Planned Parenthood clinics.^13^ In states without laws explicitly protecting minor confidentiality, clinics without Title X funding could no longer universally provide free or low-cost confidential contraceptive care to minors, apart from state-specific exceptions (eg, emancipated minors) and certain exceptions for Medicaid-enrolled youth.^14,15,16^ Although clinics that left the Title X program may have preserved subsidized care for adolescents through other means, these clinics were no longer able to leverage the unique legal protections associated with Title X funding. All clinics who left the Title X program—including Planned Parenthood affiliates—could no longer allow minors to legally consent to and be guaranteed confidential care in states where this is not protected by law.

Evidence-based Title X guidelines were reenacted November 4, 2021, and many clinics that left the program between 2019 and 2021 have since reapplied for funding.^17^ However, understanding access shifts between 2019 and 2021 has implications for confidential adolescent family planning care under future federal administrations. Athough prior research^18,19^ describes quantitative changes in program composition during the rule change, there has been no formal evaluation of the regulatory shift’s association with minors’ access to services.

The primary aim of this study was to compare the availability of confidential contraceptive services for minors before and after the Title X rule change in 2019. We hypothesized that the geographical distribution of clinics in the Title X program changed after the rule change, reducing minors’ access to confidential care at the US Census tract level. Secondarily, we aimed to identify Census tract characteristics associated with complete loss of Title X–funded services.

## Methods

### Title X Clinic Identification

This cross-sectional study was determined to be exempt from full review by our institutional review board, as the data are anonymous and publicly available. This report conforms to the Strengthening the Reporting of Observational Studies in Epidemiology (STROBE) reporting guideline for cross-sectional studies. Title X–funded entities in August 2018 and August 2020 were identified from publicly available Office of Population Affairs documents.^20,21^ August 2019 was chosen as the delineating time point on the basis of federal court decisions and the announcement by Planned Parenthood to remove clinics from the Title X program on August 19, 2019.^13,22^ To validate this definition, we reviewed the annual number of Title X clinics from 2006 to 2020 using interrupted time series analysis.^23^ The number of Title X clinics decreased before 2019 by an average of 1% per year (*b* = −68.2; 95% CI, −84.3 to −52.0). There was a significant change in clinic numbers in 2019, with a 4% decrease from 2018 to 2019 and a 20% decrease from 2019 to 2020 (*b* = −918.8; 95% CI, −935.0 to −902.7).

The Title X program distributes funds via grantees, which distribute further to subrecipients that can provide direct services and can also distribute further to service sites.^7^ We defined all subrecipients or service sites as clinics, consistent with other evaluations.^18^ Clinics located in the 50 states and the District of Columbia (DC) that left the Title X program after August 2018, joined the Title X program as of August 2020, and remained in the Title X program over this time frame were categorized as such. Duplicate clinics were excluded.

Clinics with Federally Qualified Health Center (FQHC) designation were identified using the Health Resources and Services Administration database, the US Centers for Disease Control and Prevention (CDC) National Prevention Information Network, or the clinic website.^24,25^ Planned Parenthood clinics were identified on the basis of clinic name or website. Crisis Pregnancy Centers were identified using a publicly available map or from self-identification on clinic materials.^26^ Clinics that did not fall under these categories were deemed other.

### State Laws

States were classified as either (1) universal minor confidentiality states, with the presence of state legislation allowing all minors of a certain age to consent to contraceptive services and guaranteeing the confidentiality of minors receiving these services (19 states and DC), or (2) no universal minor confidentiality states*,* with the absence of legislation allowing all minors to both consent for and confidentially receive contraceptive services, including states with no explicit laws (4 states), with laws limiting which categories of minors can access care (eg, only emancipated minors, 19 states), and with laws allowing physicians to disclose information regarding contraception to parents without minor consent (8 states) (eTable 1 in the Supplement).^15,27^ In no universal minor confidentiality states, minors’ ability to consent for and receive confidential care was no longer guaranteed by law in the absence of Title X–funded clinics. Maine, the only state with a policy change over the study period, at no point had minor confidentiality protections, and was classified as a no minor confidentiality state.^28^

### Youth Access to Confidential Contraceptive Services at the Census-Tract Level

Using the clinic address, Title X–funded clinics were geocoded with ArcGIS Pro software version 2.5.1 (Esri). Clinic locations were classified geographically using Rural-Urban Continuum Codes (RUCCs)^29^ and Census regions.^30^ RUCCs were dichotomized into urban (RUCC 1-3) and rural areas (RUCC 4-9).

Next, every Census tract was evaluated for access to legally protected confidential family planning care for minors, our primary outcome, based on 1 of 2 criteria: (1) the existence of at least 1 Title X–funded clinic within a 30-minute drive from the Census tract population-weighted centroid, or (2) location within a state or district with explicit minor confidentiality laws. We defined a 4-category variable describing changes in youth access at the census tract-level as follows: (1) lost access (access in 2018 but not 2020), (2) maintained access (access in 2018 and 2020), (3) gained access (access in 2020 but not 2018), or (4) no access throughout.

Esri’s Network Analyst extension (Esri) calculated 30-minute drive time catchments. Thirty minutes was determined as the maximum reasonable travel distance for medical care on the basis of Medicaid standards.^31^

### Covariates

We obtained estimated demographic and socioeconomic characteristics of Census tract populations from the 2015 to 2019 American Community Survey, including total population and number of minor residents aged 15 to 17 years.^32^ American Community Survey multiyear estimates allowed for increased statistical reliability in less populated areas and for smaller population subgroups. We also obtained the CDC Social Vulnerability Index (SVI), a Census tract–level measure of a community’s resilience after public health disasters.^33^ The SVI is associated with other community-level health risks, including obesity, COVID-19, and teen pregnancy.^34,35,36^

Additional Census tract characteristics assessed were population density, birth rate per 1000 female residents in the past year, number and proportion of individuals aged less than 18 years, an estimate of Medicaid-enrolled youth aged 15 to 17 years (the number of Medicaid-enrolled minors multiplied by the proportion of minors in that Census tract aged 15-17 years), and percentage of the population identifying as Black and Hispanic, respectively. Race and ethnicity were examined as social constructs given their associations with coercive reproductive care delivery, inequitable access to care due to systemic racism, and adverse adolescent sexual and reproductive health care outcomes.^37,38,39^

Less than 1% of Census tracts were missing data for each of our covariates and were excluded from the relevant univariate analysis. There were 72 620 tracts included in the analysis, accounting for approximately 324 697 728 individuals (99.96% of the population).

### Statistical Analysis

The net clinic loss was defined as the number of clinics that left the Title X program after August 2018, subtracted by the number of clinics that joined as of August 2020. Characteristics of Title X–funded clinics in 2018 and 2020 that stayed or left the Title X program and characteristics of Census tracts in which minors maintained or lost access to legally protected confidential contraceptive care were described and compared with χ^2^ tests. Two-sided significance was set at *P* < .05. An estimate of the number of youth at risk for service loss was calculated according to the total population aged 15 to 17 years in each Census tract, and stratified by Medicaid enrollment.

Univariate logistic regression evaluated associations between Census tract characteristics and odds of minors losing vs maintaining access to legally protected confidential services. This analysis excluded Census tracts where minors did not have access throughout the period (3832 tracts [5.3%]) and where minors gained access in 2020 (721 tracts [1.0%]). Continuous variables were categorized into quartiles to allow for clearer interpretation of odds ratios (ORs). We did not perform multivariate analyses because of collinearity between covariates.

We performed 2 sensitivity analyses. First, an adolescent might live in a state that protects minor confidentiality, but in an area without a clinic that provides family planning services. To evaluate this scenario, we compared minors’ access to a Title X clinic within 30 minutes at the Census tract level, irrespective of state law, before and after the rule change. Second, there are 8 states in which a minor can consent to contraceptive services, but physicians are given discretion in maintaining confidentiality. We thus repeated our analyses including these partially protective states in our definition of access to confidential care.

Statistical analysis was performed in Stata statistical software version 16 (StataCorp) between January and December 2021. Our data and code are publicly available.^40^

## Results

In 2018, there were 4466 Title X–funded clinics. After the rule change, 1743 clinics left the program (39.0%), 109 clinics were identified as duplicates, and 494 new clinics entered the program, resulting in a net loss of 1249 clinics (28.0%) (Table 1 and eFigure in the Supplement). There was a net loss of 224 FQHCs, and a substantially larger proportion of FQHCs in the program after the rule change (37.2%) than before (31.7%). Almost all Planned Parenthood sites left the program after the rule change (486 sites [99.0%]), corresponding to 11.2% of the Title X program. Eight crisis pregnancy centers joined the program after the rule change.

**Table 1.** Characteristics of Title X–Funded Clinics Before and After the Title X Rule Change

Clinic characteristic	Clinics, No. (%)^a^			*P* value^b^
	2018	2020	Net change	
No.	4343^c^	3094^d^	−1249 (28.0)	NA
Affiliation				
Crisis pregnancy center	0	8 (0.3)	8 (100)	.001
Federally Qualified Health Center	1375 (31.7)	1151 (37.2)	−224 (16.3)	<.001
Planned Parenthood	486 (11.2)	5 (0.2)	−481 (99.0)	<.001
Other	2482 (57.2)	1930 (62.4)	−552 (22.2)	<.001
Population density^e^				
Urban	2793 (64.3)	1870 (60.4)	−923 (33.0)	<.001
Rural	1550 (35.7)	1224 (39.6)	−326 (21)	
Geographical region				
Northeast	781 (18.0)	313 (10.1)	−468 (60.0)	<.001
Midwest	716 (16.5)	499 (16.1)	−217 (30.3)	.68
South	1861 (42.9)	1688 (54.6)	−173 (9.3)	<.001
West	985 (22.7)	594 (19.2)	−391 (39.7)	<.001
State minor confidentiality laws				
State has protected minor confidentiality	2214 (51.0)	1598 (51.6)	−616 (27.8)	.57
Does not have protected minor confidentiality	2129 (49.0)	1496 (48.4)	−633 (29.7)	

Abbreviation: NA, not applicable.

^a^ Percentages may not sum to 100% because of rounding.

^b^ All differences were compared using χ^2^ testing.

^c^ Excludes 123 clinics that were found to be duplicates.

^d^ Excludes 14 clinics that were found to be duplicates.

^e^ Rural-Urban Continuum Codes 1 to 3 were classified as urban, and codes 4 to 9 were classified as rural.

Although the total number of Title X–funded clinics decreased throughout the US, a larger proportion of Title X–funded clinics were distributed in rural areas after the rule change (1550 clinics [35.7%] in 2018 vs 1224 clinics [39.6%] in 2020) and in the South (1861 clinics [42.9%] in 2018 vs 1688 clinics [54.6%] in 2020). There was a significantly smaller proportion of clinics in the Northeast (781 clinics [18.0%] in 2018 vs 313 clinics [10.1%] in 2020) or West (985 clinics [22.7%] in 2018 vs 594 clinics [19.2%] in 2020). We found no significant difference in the proportion of Title X–funded clinics located in states without minor confidentiality laws after the rule change (2129 clinics [49%] in 2018 vs 1496 clinics [48.4%] in 2020). There was a 29.7% decrease (633 clinics) in the number of Title X–funded clinics in states without minor confidentiality laws.

### Youth Access to Confidential Care at the Census Tract Level

**Figure 1.**
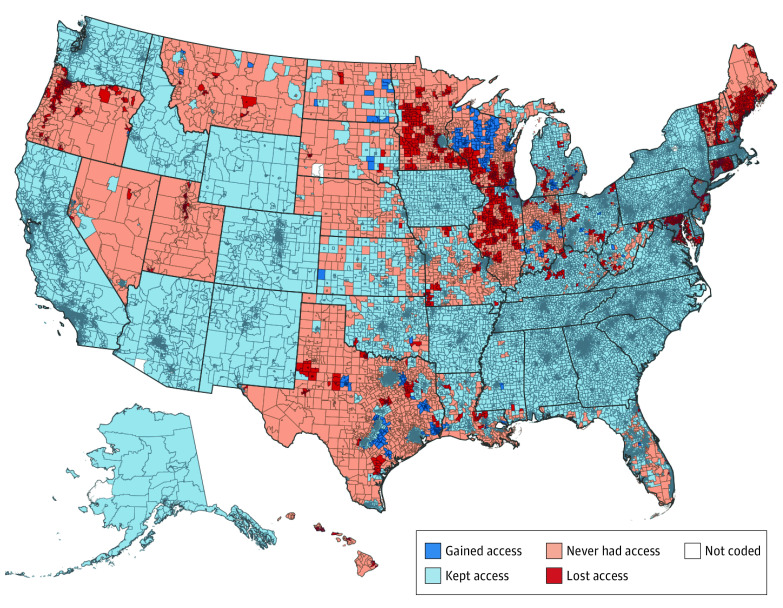
Access to Confidential Family Planning Services for Minors After the Title X Rule Change, by US Census Tract

Of the 72 760 Census tracts evaluated, minors living in 6299 (8.7%) lost access to confidential contraceptive services after the Title X rule change, corresponding to 933 649 youth aged 15 to 17 years (Figure 1 and Table 2). An estimated 642 850 of these youth (68.9%) were not Medicaid enrolled and, therefore, did not maintain confidential access through Medicaid statutes.

**Table 2.** Access to Confidential Services for Minors After the Title X Rule Change, by US Census Tract

US Census tract characteristics^b^	Confidential care for minors after the Title X rule change, Census tracts, No. (%)^a^		Odds of losing access to confidential minor services, OR (95% CI)^c^
	Kept access	Lost all access	
All Census tracts^d^	61 063 (83.9)	6299 (8.7)	NA
Geography			
Population density^e^			
Urban	52 924 (86.7)	5271 (83.7)	1 [Reference]
Rural	8139 (13.3)	1028 (16.3)	1.27 (1.18-1.36)
Census region			
Northeast	12 069 (19.8)	1153 (18.3)	1 [Reference]
Midwest	11 740 (19.2)	2706 (43.0)	2.41 (2.24-2.60)
South	23 241 (38.1)	1003 (15.9)	0.45 (0.41-0.49)
West	14 013 (23.0)	1437 (22.8)	1.07 (1.0-1.16)
SVI percentile			
SVI in bottom quartile (least at risk)	14 824 (24.5)	2049 (32.7)	1 [Reference]
Second quartile	14 708 (24.3)	1700 (27.1)	0.84 (0.78-0.90)
Third quartile	15 063 (24.9)	1394 (22.2)	0.67 (0.62-0.72)
SVI in top quartile (most at risk)	15 978 (26.4)	1127 (18.0)	0.51 (0.47-0.55)
Population characteristics			
Race			
Proportion Black in bottom quartile (<0.90%)	13 025 (21.4)	2248 (35.8)	1 [Reference]
Second quartile	15 033 (24.7)	1824 (29.1)	0.70 (0.66-0.75)
Third quartile	16 046 (26.4)	1230 (19.6)	0.44 (0.41-0.48)
Proportion Black in top quartile (>15.0%)	16 682 (27.4)	976 (15.6)	0.34 (0.31-0.37)
Ethnicity			
Proportion Hispanic in bottom quartile (<2.8%)	14 287 (23.5)	1778 (28.3)	1 [Reference]
Second quartile	14 759 (24.3)	1965 (31.3)	1.07 (1.00-1.15)
Third quartile	15 441 (25.4)	1615 (25.7)	0.84 (0.78-0.90)
Proportion Hispanic in top quartile (>20.9%)	16 299 (26.8)	920 (14.7)	0.45 (0.42-0.49)
Birth rate			
Birth rate in bottom quartile (<20/1000 population)	15 286 (25.2)	1472 (23.5)	1 [Reference]
Second quartile	14 804 (24.4)	1659 (26.4)	1.16 (1.08-1.25)
Third quartile	15 464 (25.4)	1674 (26.6)	1.12 (1.04-1.21)
Birth rate in top quartile (>76/1000 population)	15 232 (25.1)	1473 (23.5)	1.00 (0.93-1.08)
Age distribution			
Proportion aged <18 y in bottom quartile (<19.5%)	15 403 (25.3)	1525 (24.3)	1 [Reference]
Second quartile	15 045 (24.8)	1686 (26.9)	1.13 (1.05-1.22)
Third quartile	14 947 (24.6)	1591 (25.3)	1.08 (1.00-1.16)
Proportion aged <18 y in top quartile (>27.2%)	15 391 (25.3)	1476 (23.5)	0.97 (0.90-1.04)
State minor confidentiality laws			
Universal minor confidentiality law	34 452 (56.4)	0	NA
No universal minor confidentiality law	26 611 (43.6)	6299 (100)	NA

Abbreviations: NA, not applicable; OR, odds ratio; SVI, Social Vulnerability Index.

^a^ Percentages may not sum to 100% because of rounding.

^b^ All observations had less than 1% missing data.

^c^ Calculated using univariate logistic regression.

^d^ The total number of Census tracts includes the 6.4% of census tracts that never had access to confidential services (4669 tracts) and the 1.0% that gained access after the Title X rule change (729 tracts).

^e^ Rural-Urban Continuum Codes 1 to 3 were classified as urban, and codes 4 to 9 were classified as rural.

**Figure 2.**
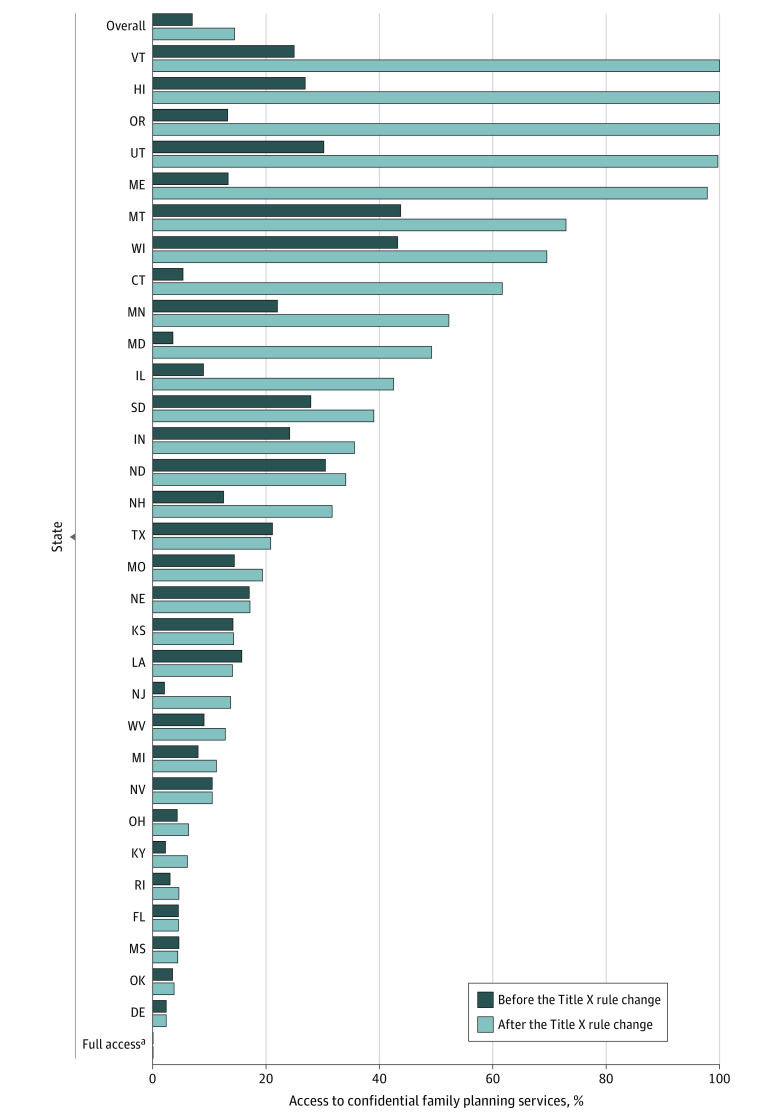
Percentage of Youth Aged 15 to 17 Years Without Access to Confidential Family Planning Services Before and After the Title X Rule Change, by State ^a^Youth in the following 20 states and district had full access to confidential care both before and after the Title X rule change: Alaska, Alabama, Arkansas, Arizona, California, Colorado, District of Columbia, Georgia, Iowa, Idaho, Massachusetts, North Carolina, New Mexico, New York, Pennsylvania, South Carolina, Tennessee, Virginia, Washington, and Wyoming.

Overall, as of 2020, there were 10 968 tracts (15.1%) in which minors did not have access to confidential contraceptive services, affecting 1 811 818 youth aged 15 to 17 years. In 15 states, more than 25% of youth aged 15 to 17 years did not have legally protected confidentiality when accessing contraceptive services, compared with 6 states in 2018 (Figure 2).

**Figure 3.**
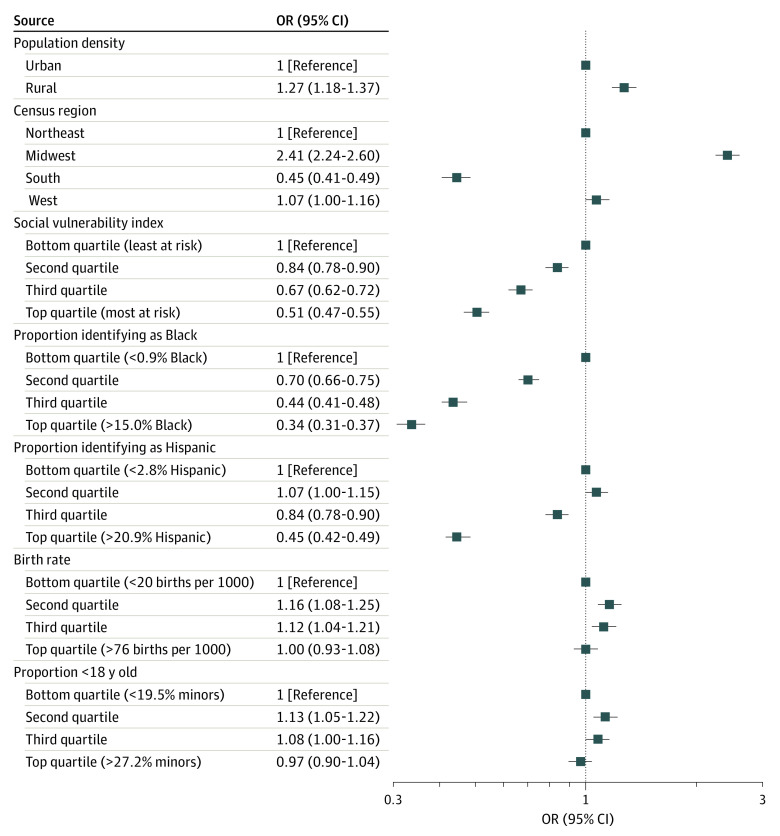
Odds of Losing Access to Confidential Minor Services After the Title X Rule Change, by Selected US Census Tract Characteristics OR indicates odds ratio.

Compared with minors living in the Northeast, those living in the Midwest had higher odds of losing access to legally protected confidential services (OR, 2.41; 95% CI, 2.24-2.60) and those in the South had lower odds (OR, 0.45; 95% CI, 0.41-0.49) (Figure 3 and Table 2). Minors in rural areas had higher odds of losing access compared with those in urban areas (OR, 1.27; 95% CI, 1.18-1.36).

Compared with minors living in Census tracts in the lowest SVI quartile, minors in the highest SVI quartile Census tracts had 49% decreased odds of losing access to protected confidential care (OR, 0.51; 95% CI, 0.47-0.55) (Figure 3 and Table 2). Minors living in Census tracts with higher proportions of Black and Hispanic individuals also had lower odds of losing access to confidential care compared with those in tracts with the lowest proportions of Black (OR, 0.34; 95% CI, 0.31-0.37) and Hispanic (OR, 0.45; 95% CI, 0.42-0.49) individuals.

Our sensitivity analysis identified an additional 6132 Census tracts (575 rural and 5557 urban) in which minors lost their only accessible Title X clinic after the rule change, although these tracts were in permissive states and therefore maintained access to care by study definition. In expanding our definition of confidential access to include all states in which a minor can consent for care, including those states without assurances of legally protected confidentiality, we identified minors in 3954 Census tracts (5.4%) who lost access to confidential care after the Title X rule change, with characteristics similar to those in our principal analysis (eTable 2 in the Supplement).

## Discussion

This cross-sectional study identified that there have been critical losses in access to confidential contraceptive services for youth since the 2019 Title X rule change. As of 2020, more than 1.8 million adolescents aged 15 to 17 years lived in a Census tract without access to legally protected confidential family planning care. More than half of these youth lost access to services after the 2019 Title X rule change. Thirty-nine percent of clinics in the Title X program left, including 99% of Planned Parenthood clinics. Eight crisis pregnancy centers, facilities that intentionally limit access to nondirective pregnancy counseling and certain forms of contraception, joined the program after the rule change.^41^

Census tracts in which minors lost access to confidential contraceptive services after the rule change had more socioeconomic advantage than tracts maintaining access. Socioeconomic disadvantage is an independent risk factor for teen pregnancy^42^ and sexually transmitted infection acquisition.^43^ An estimated 25% of reproductive-aged women are not using their preferred contraceptive method because they cannot afford it.^44^ The relative affluence of the communities that lost access to confidential care may partially buffer the adverse outcomes associated with the rule change.

Census tracts in which minors lost access to confidential services were also more likely to have a lower proportion of Black and Hispanic individuals. This finding may be partially explained by the distribution of Title X–funded FQHCs, as FQHCs serve an outsized role in providing care for minoritized populations and were less likely to leave the Title X program in our analysis.^45^ However, even before the Title X rule change, Black and Hispanic youth faced inequitable access to evidence-based family planning care, a consequence of historical and present-day racism in sexual and reproductive health services.^38^ As the Title X program rebuilds, ensuring equal distribution of funds to support high-quality family planning care for historically marginalized individuals, such as those identifying as Black, Indigenous, and/or Hispanic, has been prioritized.^17^ Notably, our data suggest that simply reinstating the clinics that left the program may not adequately achieve this goal.

We found a 9% decrease in the number of Title X–funded clinics in states without minor confidentiality laws after the Title X rule change. This reduction was most notable in the Midwest, where minors had more than 2-fold increased odds of living in a Census tract without protected confidential services after the rule change. Driving this finding is the current legal landscape in the Midwest, in which 11 of 12 states (92%) do not have state laws protecting minor consent and confidentiality.^27^ Further research evaluating adolescents’ experiences seeking confidential sexual and reproductive health services in this region may help inform state policy going forward.

The reinstatement of evidence-based Title X guidelines in November 2021 was a crucial first step in maintaining access to comprehensive health care for adolescents.^17^ Our data highlight the additional need for states to enact laws explicitly permitting all adolescents to consent for, and be ensured confidentiality in, obtaining sexual and reproductive health services. These legal protections are in line with recommendations from major medical societies,^3,5^ allow adolescents to receive higher quality care irrespective of Title X funding, and protect youth from future risks of politically enacted Title X restrictions that do not align with evidence-based practice.

### Limitations

This study is limited to evaluating changes in Title X clinic distribution and is unable to explore changes in minors’ ability to access care in practice. Clinic infrastructure, clinician beliefs, and clinician knowledge of state laws may limit the availability of confidential services for minors, even in states where confidential care is permitted.^46^ However, although these factors may be overcome by individual clinics prioritizing evidence-based family planning care, the legal barriers identified in our research cannot be superseded. We are also unable to prove the causality of the rule change on the decision of a clinic to leave or join the Title X program, although we documented a significant change in trend starting in 2019.

Our estimates of the number of adolescents potentially impacted by these changes are conservative and may underestimate actual service loss. We only included 15- to 17-year-olds in our calculations because of available age categories in Census data. With respect to access to care, the 30-minute drive time definition may be prohibitively far for many adolescents. Adolescents in rural areas within permissive states may not feasibly have access to care, including those in the 575 rural Census tracts identified in our sensitivity analysis. Additionally, we included 8 crisis pregnancy centers in our analysis, which are entitled to confidentiality protections but may not provide evidence-based family planning care.

Exemptions exist allowing Medicaid-enrolled youth to consent to confidential contraceptive care in states without permissive laws; hence, we reported estimates including and excluding this population. Medicaid exemptions require an adolescent to provide health insurance information, rely on clinical staff awareness of Medicaid-associated confidentiality protections, and do not universally restrict itemized accounts of services being sent to homes, all of which may limit the practical utility of this exemption.^47^

## Conclusions

In this evaluation of the association of the 2019 Title X rule change with minor access to reproductive health care, we document significant shifts in the composition of the Title X program and a loss of access to confidential family planning services for an estimated 933 649 adolescents living in 8.7% of US Census tracts after the rule change was enacted. Adolescents living in rural areas or the Midwest disproportionately lost access to confidential services. Our findings highlight how federal policy change may change adolescent access to confidential reproductive health care.

## Notes

Supplement. **eTable 1. **Legal Status of Confidential Contraceptive Services for Minors by State, 2018 to 2020 **eTable 2. **Access to Minor Consent for Contraceptive Services After the Title X Rule Change, by US Census Tract **eFigure. **Title X-Funded Clinics Before and After the 2019 Title X Rule Change
