# Supplementary material for: Adolescent Access to Federally Funded Clinics Providing Confidential Family Planning Following Changes to Title X Funding Regulations
Source: JAMA Netw Open. 2022 Jun 17;5(6):e2217488. doi: 10.1001/jamanetworkopen.2022.17488 (PMC9206194; doi:10.1001/jamanetworkopen.2022.17488)
Supplement: Supplement. — eTable 1. Legal Status of Confidential Contraceptive Services for Minors by State, 2018 to 2020 eTable 2. Access to Minor Consent for Contraceptive Services After the Title X Rule Change, by US Census Tract eFigure. Title X-Funded Clinics Before and After the 2019 Title X Rule Change [file jamanetwopen-e2217488-s001.pdf]

Supplemental Online Content

Krass P, Tam V, Min J, et al. Adolescent access to federally funded clinics providing confidential family planning following changes to Title X funding regulations. *JAMA Netw Open*. 2022;5(6):e2217488. doi:10.1001/jamanetworkopen.2022.17488

eTable 1. Legal Status of Confidential Contraceptive Services for Minors by State, 2018 to 2020
eTable 2. Access to Minor Consent for Contraceptive Services After the Title X Rule Change, by US Census Tract
eFigure. Title X-Funded Clinics Before and After the 2019 Title X Rule Change

This supplemental material has been provided by the authors to give readers additional information about their work.

© 2022 Krass P et al. *JAMA Network Open.*

eTable 1. Legal Status of Confidential Contraceptive Services for Minors by State, 2018 to 2020

State	Access to Confidential Contraceptive Services for Minors
AK	Universal Minor Confidentiality
AL	Universal Minor Confidentiality
AR	Universal Minor Confidentiality
AZ	Universal Minor Confidentiality
CA	Universal Minor Confidentiality
CO	Universal Minor Confidentiality
CT	No Universal Minor Consent or Confidentiality
DC	Universal Minor Confidentiality
DE	Minor Consent but No Confidentiality
FL	No Universal Minor Consent or Confidentiality
GA	Universal Minor Confidentiality
HI	Minor Consent but No Confidentiality
IA	Universal Minor Confidentiality
ID	Universal Minor Confidentiality
IL	No Universal Minor Consent or Confidentiality
IN	No Universal Minor Consent or Confidentiality
KS	No Universal Minor Consent or Confidentiality
KY	Minor Consent but No Confidentiality
LA	No Universal Minor Consent or Confidentiality
MA	Universal Minor Confidentiality
MD	Minor Consent but No Confidentiality
ME	Minor Consent but No Confidentiality[a]
MI	No Universal Minor Consent or Confidentiality
MN	Minor Consent but No Confidentiality
MO	No Universal Minor Consent or Confidentiality
MS	No Universal Minor Consent or Confidentiality
MT	Minor Consent but No Confidentiality
NC	Universal Minor Confidentiality
ND	No Policy In Place
NE	No Universal Minor Consent or Confidentiality
NH	No Universal Minor Consent or Confidentiality
NJ	No Universal Minor Consent or Confidentiality
NM	Universal Minor Confidentiality
NV	No Universal Minor Consent or Confidentiality
NY	Universal Minor Confidentiality
OH	No Policy In Place
OK	No Universal Minor Consent or Confidentiality
OR	Minor Consent but No Confidentiality
PA	Universal Minor Confidentiality
RI	No Policy In Place
SC	Universal Minor Confidentiality
SD	No Universal Minor Consent or Confidentiality
TN	Universal Minor Confidentiality
TX	No Universal Minor Consent or Confidentiality
UT	No Universal Minor Consent or Confidentiality
VA	Universal Minor Confidentiality
VT	No Universal Minor Consent or Confidentiality
WA	Universal Minor Confidentiality
WI	No Policy In Place
WV	No Universal Minor Consent or Confidentiality
WY	Universal Minor Confidentiality

[a]Prior to 2019, Maine was classified as “No Universal Minor Consent or Confidentiality.” No other states had a policy change over this time frame.

© 2022 Krass P et al. *JAMA Network Open.*

eTable 2. Access to Minor Consent for Contraceptive Services After the Title X Rule Change, by US Census Tract

Census Tract Characteristics[a]	Access to Minor Consent after the Title X Rule Change		Odds of losing access to minor consent (95% CI)[b]
	Kept access	*Lost all access*	
N (% of all census tracts[c])	64,180 (88.2%)	3,954 (5.4%)	
**Geography[d], n(%)**			
Population Density			
Urban	55,142 (85.9%)	3,351 (84.7%)	Reference
Rural	9,038 (14.1%)	603 (15.3%)	1.1 (1.00-1.20)
Census Region			
Northeast	12,342 (19.2%)	880 (22.2%)	Reference
Midwest	12,396 (19.3%)	2,361 (59.7%)	2.67 (2.46-2.90)
South	24,062 (37.5%)	281 (7.1%)	0.16 (0.14-0.19)
West	15,380 (24.0%)	432 (10.9%)	0.39 (0.35-0.44)
Social Vulnerability Index (SVI) Percentile			
SVI in bottom quartile (least at risk)	15,682 (24.6%)	1,369 (34.8%)	Reference
Second quartile	15,649 (24.6%)	1,021 (25.9%)	0.75 (0.69-0.81)
Third quartile	15,855 (24.9%)	830 (21.1%)	0.60 (0.55-0.66)
SVI in top quartile (most at risk)	16,474 (25.9%)	717 (18.2%)	0.50 (0.45-0.55)
**Population Characteristics, n(%)**			
Race			
Proportion Black in bottom quartile (<0.90%)	14,524 (22.7%)	1,295 (32.9%)	Reference
Second quartile	15,803 (24.7%)	1,201 (30.5%)	0.85 (0.79-0.93)
Third quartile	16,467 (25.8%)	854 (21.7%)	0.58 (0.53-0.64)
Proportion Black in top quartile (>15.0%)	17,087 (26.8%)	592 (15.0%)	0.39 (0.35-0.43)
Ethnicity			
Proportion Hispanic in bottom quartile (<2.8%)	15,423 (24.1%)	993 (25.2%)	Reference
Second quartile	15,826 (24.8%)	1,159 (29.4%)	1.14 (1.04-1.24)
Third quartile	16,158 (25.3%)	1,022 (25.9%)	0.98 (0.90-1.07)
Proportion Hispanic in top quartile (>20.9%)	16,474 (25.8%)	768 (19.4%)	0.72 (0.66-0.80)
Birthrate			
Birthrate in bottom quartile (<20 per 1000)	16,064 (25.2%)	924 (23.4%)	Reference
Second quartile	16,641 (24.5%)	1,061 (26.9%)	1.18 (1.08-1.29)
Third quartile	16,274 (25.5%)	1,035 (26.3%)	1.11 (1.01-1.21)
Birthrate in top quartile (>76 per 1000)	15,902 (24.9%)	922 (23.4%)	1.01 (0.92-1.07)
Age Distribution			
Proportion aged <18 in bottom quartile (<19.5%)	16,279 (25.5%)	879 (22.3%)	Reference
Second quartile	15,933 (24.9%)	1,009 (25.6%)	1.17 (1.07-1.29)
Third quartile	15,755 (24.7%)	988 (25.1%)	1.16 (1.06-1.28)
Proportion aged <18 in top quartile (>27.2%)	15,914 (24.9%)	1,066 (27.0%)	1.24 (1.13-1.36)
**State Minor Consent Laws, n(%)**			
Allows universal minor consent	40,216 (62.7%)	0 (0.0%)	-
Does not allow universal minor consent	23,964 (37.3%)	3,954 (100%)	-

a. All observations with <1% missing data.
b. Calculated using univariate logistic regression.
c. The total number of census tracts includes the 5.3% of census tracts who never had access to minor consent (n=3,832) and the 1.1% who gained access, including those in Maine where state law changed over this time frame to allow minor consent (n=794).
d. Rural-Urban Continuum Codes (RUCCs) 1-3 classified as urban, 4-9 classified as rural.
e. Percentages may not sum to 100% due to rounding.

© 2022 Krass P et al. *JAMA Network Open.*

eFigure 1. Title X-Funded Clinics Before and After the 2019 Title X Rule Change

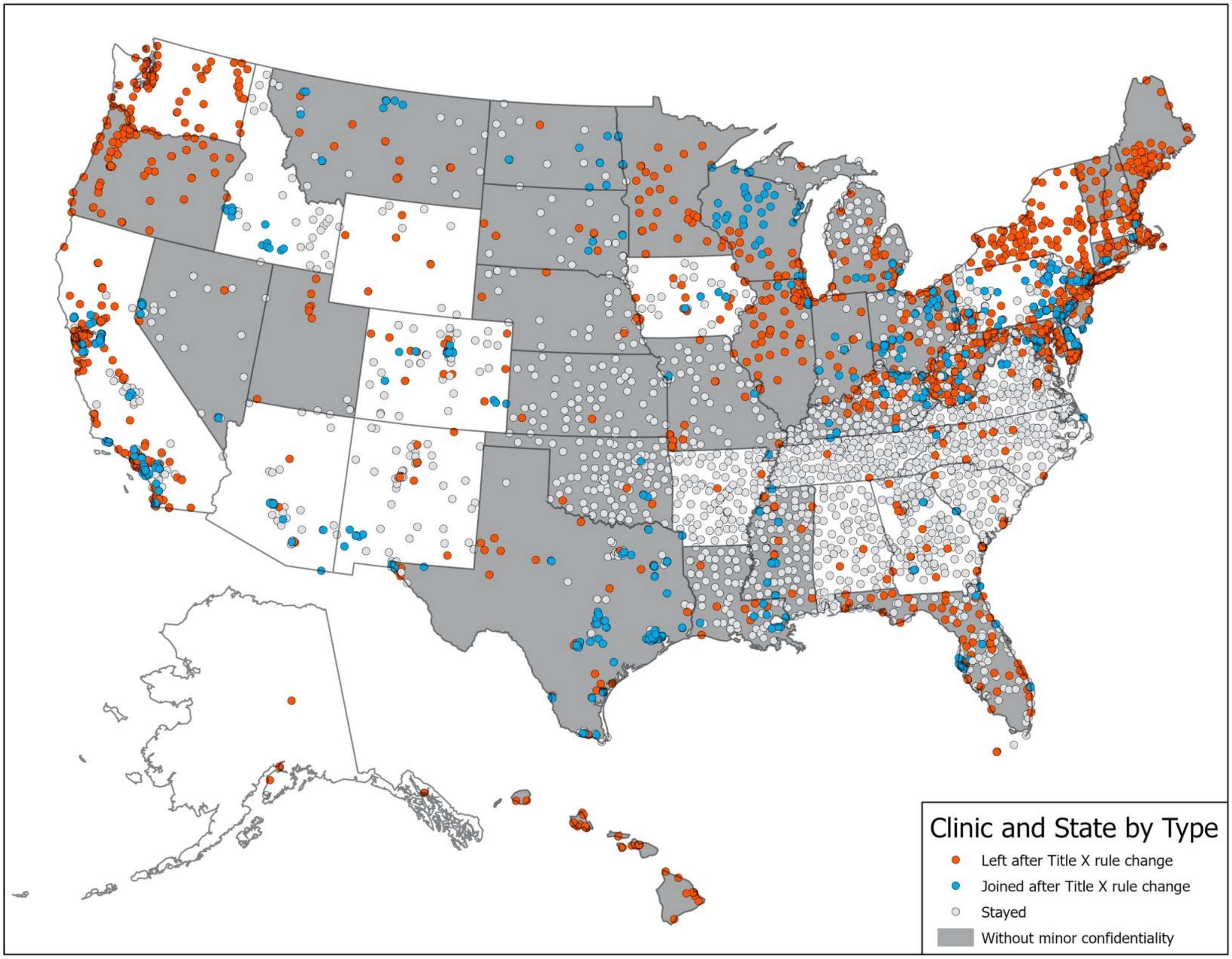

© 2022 Krass P et al. *JAMA Network Open.*
